# Artificial intelligence readiness and its influencing factors among newly qualified nurses: a cross-sectional study

**DOI:** 10.3389/fmed.2026.1753024

**Published:** 2026-01-28

**Authors:** Qianqian Yang, Min Zhao, Linlin Yang, Xiaobing Wang, Chunling Yang

**Affiliations:** 1 Department of Nursing, Liaocheng People’s Hospital, Medical School of Liaocheng University, Liaocheng, Shandong, China; 2 School of Nursing and Rehabilitation, Shandong University, Jinan, Shandong, China; 3 School of Nursing, Shandong First Medical University, Shandong Academy of Medical Sciences, Tai'an, Shandong, China; 4 Department of Nursing, Liaocheng People’s Hospital, Liaocheng, Shandong, China; 5 Obstetrics Department, Liaocheng People’s Hospital, Liaocheng, Shandong, China

**Keywords:** artificial intelligence readiness, newly qualified nurses, perceived barriers, perceived ease of use, technology acceptance model

## Abstract

**Introduction:**

This study investigated the artificial intelligence (AI) readiness of newly qualified nurses and identified potential influencing factors. The technology acceptance model was extended by including perceived barriers to provide a comprehensive understanding of AI adoption in clinical practice.

**Methods:**

This cross-sectional study was conducted across four tertiary grade A hospitals in Shandong Province in August and September 2022. Using convenience sampling, 329 newly qualified nurses with 1–3 years of clinical experience were surveyed. Data were collected using several instruments: a demographic characteristics questionnaire, the Readiness to Adopt AI in Nursing Practice Scale, the Perceived Usefulness in Nursing Practice Scale, the Perceived Ease of Use in Nursing Practice Scale, and the Perceived Barriers to Accessing AI Technology Scale. Data analysis, including descriptive statistics, correlation analysis, and multiple linear regression, was performed using SPSS 27.0.

**Results:**

Newly qualified nurses’ AI readiness was moderate (M = 9.85, SD = 1.97). Multiple linear regression identified three significant factors associated with AI readiness: perceived ease of use (*β* = 0.211, *p* = 0.006), prior AI training (*β* = 0.23, *p* < 0.001), and awareness of AI in nursing practice (*β* = 0.201, *p* = 0.018). Although perceived barriers did not significantly predict readiness in regression analysis, they were widely prevalent in clinical practice, with a lack of AI knowledge and limited computer skills reported as common obstacles.

**Discussion:**

The readiness of newly qualified nurses for AI is influenced by multiple factors. Awareness of AI plays a crucial role, in addition to perceived ease of use and prior AI training. Although perceived barriers did not show a significant relationship with readiness, practical challenges, such as knowledge gaps and limited computer skills, require attention. Enhancing AI training, improving system usability, and ensuring adequate time and resource support are essential to strengthen AI application capabilities among newly qualified nurses.

## 1 Introduction

Artificial intelligence (AI), a core technology that drives digital transformation, has profoundly affected diverse fields, including education, management, and finance, with an increasingly prominent role in healthcare (1). Recently, the use of AI-based healthcare has experienced significant growth. The application of AI in healthcare, such as auxiliary diagnosis (2), disease risk prediction (3), and personalized treatment algorithms (4), has enhanced diagnostic accuracy, improved treatment efficiency, and optimized clinical workflows (5). AI has also demonstrated considerable potential to revolutionize nursing practice by supporting clinical decision-making, aiding physical rehabilitation, and enabling intelligent health education (6–8). Despite its potential, integrating AI into nursing poses challenges such as data privacy, security concerns, and data quality limitations that must be acknowledged and addressed (9, 10). However, as AI adoption in nursing practice increases, research on newly qualified nurses’ readiness to use AI tools in professional practice remains scarce. Implementing AI without first assessing these nurses’ readiness could adversely affect their practice quality and professional development focus.

Newly qualified nurses, defined as those in their first 1 to 3 years of professional practice after graduation (11), represent a critical and distinct group. Compared to more senior nurses, they generally have stronger digital literacy and a higher degree of technological acceptance (12, 13). Furthermore, as they transition from students to professional nurses, their work habits and clinical reasoning are still developing, granting them greater adaptability (14, 15). However, based on the “Novice to Expert” framework (16), newly qualified nurses are typically in the early stages of skill development and rely heavily on rules and guidelines to inform their actions (17). This stage is characterized by limited experience and a higher dependency on structured support. Consequently, their readiness to adopt new technologies such as AI may be influenced by their ability to understand and apply these technologies in the context of their developing clinical skills. Moreover, as future core members of the nursing workforce, their attitudes toward and acceptance of AI technology will profoundly shape future nursing practice models (18). If this group is not adequately prepared for AI adoption, they may miss opportunities to improve care efficiency, face increased workloads, and struggle to adapt to AI-driven clinical environments, ultimately hindering broader AI integration in nursing (19). Therefore, assessing the readiness of newly qualified nurses to use AI technologies in their study and identifying possible influencing factors are essential tasks. Previous studies on AI readiness in nursing have primarily focused on nursing students and educators. For example, research has shown that student nurses’ readiness for AI is associated with their attitudes, perceived usefulness, social influence, and knowledge levels (1). Among student nurses, these factors may be shaped by academic curricula and peer interactions (20). In contrast, newly qualified nurses have completed their formal education and entered clinical practice. Their readiness to adopt AI depends on its successful deployment and reliable functioning in the intricate context of clinical practice.

In this context, this study uses the technology acceptance model (TAM) as its core theoretical framework. Proposed by Davis in 1989, this well-established model explains that an individual’s readiness to adopt new technologies is governed by two key factors: perceived usefulness (how useful a system is perceived to be) and perceived ease of use (how effortlessly it is perceived to operate) (21). However, considering highly complex clinical environments, the adoption of new technologies among healthcare professionals is influenced not only by perceived usefulness and perceived ease of use but also by factors such as knowledge, skills, and organizational support (22). For newly qualified nurses, who may encounter practical obstacles in practice, such as a lack of time and AI knowledge, their perceptions of these barriers are crucial for shaping their willingness to adopt new technologies. (23). Therefore, this study introduces the concept of ‘perceived barriers’ as a supplementary factor for the TAM. This concept derives from the ‘perceived behavioral control’ dimension of the theory of planned behavior (24), which refers to the perception of the ease or difficulty of performing a behavior. Further supporting this view, other studies have noted that perceived barriers are crucial factors that influence healthcare workers’ readiness for AI (1). Therefore, this study augments the traditional TAM variables of perceived usefulness and perceived ease of use by integrating ‘perceived barriers’ as a third key variable. By systematically assessing AI readiness among newly qualified nurses and identifying its influencing factors, this study aimed to provide targeted empirical evidence for hospital administrators, nursing educators, and AI developers. Furthermore, by integrating perceived barriers into the classic TAM to better reflect clinical realities, this study explored the model’s applicability to complex healthcare environments, offering a new theoretical perspective and analytical framework for future studies. The findings may also contribute to improving the user experience of AI products, thereby enhancing AI acceptance and competency among newly qualified nurses and supporting the effective incorporation of AI into clinical nursing workflows.

Considering the above, this study has two specific research objectives:

1. Assess the current level of AI readiness among newly qualified nurses.
2. Explore the factors influencing readiness for AI adoption and examine the perceived barriers to using AI technologies in clinical practice, including their severity and prevalence.

## 2 Methods

### 2.1 Research design

This study used a cross-sectional design. Data were collected in Shandong Province, China, in August and September 2022.

### 2.2 Sampling and participants

The required sample size was determined with reference to Kendall (25), who recommended including 10–20 participants per independent variable. The study initially included 14 independent variables, comprising 10 demographic characteristics and 4 core variables (readiness to adopt AI, perceived usefulness, perceived ease of use, and perceived barriers). Accordingly, the minimum theoretical sample size ranged from 140 to 280 participants. The calculated sample size was increased by 15% to account for potential invalid or incomplete questionnaires. A total of 350 newly qualified nurses were recruited, and 21 questionnaires were excluded because of excessively short completion time (less than 5 min), missing information, or failure to pass the attention-check item (e.g., selecting “D” for the designated question). Ultimately, 329 valid questionnaires were retained for the final data analysis, with an effective response rate of 94.0%. Convenience sampling was used to recruit newly qualified nurses from four tertiary A-level hospitals (the highest level in China’s healthcare system) in Shandong Province. Participants were included if they (1) held a nurse qualification certificate, (2) voluntarily consented to participate, and (3) had accumulated 1 to 3 years of clinical nursing experience after graduation (11). The exclusion criteria were as follows: (1) having more than 3 years of nursing experience, (2) not being employed full-time (e.g., part-time staff), (3) engagement in further training or advanced studies during the survey period, and (4) being temporarily not in a nursing position for reasons such as illness, maternity leave, or personal leave.

### 2.3 Measures

#### 2.3.1 Demographic characteristics questionnaire

Participants’ demographic information was collected using a self-developed questionnaire. The collected data included age, gender, educational level, years of working experience, clinical department, prior training in AI, duration of Internet usage, self-rated understanding of AI technologies, self-rated familiarity with AI technologies, and awareness of AI in nursing.

#### 2.3.2 Readiness to adopt AI in nursing scale

This scale was initially developed by Ayanwale et al. (26) and subsequently adapted by Labrague (1). It comprises three items: (1) “I have adequate understanding and knowledge of AI in my nursing practice”; (2) “I have the opportunity to acquire the technical skills, resources, and practical applications related to AI”; and (3) “My institution supports the integration of AI into clinical care.” The items were rated on a 5-point Likert scale (1 = completely disagree to 5 = completely agree), with higher scores reflecting greater readiness. Consistent with the approach commonly adopted in previous research, the theoretical midpoint of the scale was used as a reference for interpreting scores (27). Previous research has demonstrated the scale’s satisfactory reliability and validity, reporting a Cronbach’s *α* of 0.92 (1). The scale underwent a rigorous translation and back-translation procedure to ensure linguistic and conceptual fit within the Chinese nursing context (28). Two postgraduate nurses with more than 5 years of working experience and proficiency in English independently translated the original English version into Chinese. Subsequently, two other postgraduate nurses, who were unfamiliar with the original scale, translated the Chinese version back into English. The research team rigorously compared the back-translated English version with the original scale. Finally, we conducted a pilot survey with 30 newly qualified nurses to ensure clear semantics and cultural relevance. In this study, Cronbach’s *α* for the scale was 0.735, indicating acceptable internal consistency.

#### 2.3.3 Perceived usefulness of AI in nursing practice scale

The perceived usefulness of AI in nursing practice was evaluated using a 6-item scale originally developed by Davis (21). The scale comprises the following items: (1) “Using AI at work enables me to complete tasks more quickly,” (2) “Using AI can enhance my work performance,” (3) “Utilizing AI at work will enhance my productivity,” (4) “Using AI will enhance my work efficiency,” (5) “Using AI will make my work much easier,” and (6) “I think AI to be very useful in my work.” Participants’ perceptions were measured on a 5-point Likert scale (1 = strongly disagree to 5 = strongly agree), with higher scores indicating greater perceived usefulness. The scale has demonstrated satisfactory reliability and validity in prior research (1, 29), and in the Chinese context, Cronbach’s *α* was reported as 0.944 (30). In this study, Cronbach’s α was 0.966, further supporting the suitability of this scale for measuring perceived usefulness among nurses in China.

#### 2.3.4 Perceived ease of use of AI in nursing practice scale

Perceived ease of use of AI was assessed using a 6-item scale developed by Davis (21), designed to assess acceptance and adoption of information technology systems in workplace settings, and widely applied in the field of AI. This 5-point Likert scale (1 = strongly disagree, 5 = strongly agree) measures perceived ease of use through the following items: (1) “Learning to operate AI is very easy for me,” (2) “I think it very easy to make the AI do what I want it to do,” (3) “My interaction with AI is clear and understandable,” (4) “I think AI is very flexible in terms of interaction,” (5) “It is very easy to master the use of AI,” and (6) “I think AI is very easy to use.” Higher scores indicate greater perceived ease of use in nursing practice. The scale has demonstrated good reliability in previous studies (31), and the Cronbach’s *α* score was 0.919 in a Chinese population (30). In this study, Cronbach’s α score was 0.950.

#### 2.3.5 Barriers to accessing AI technology scale

Perceived barriers to accessing AI technology were evaluated using a scale developed by Ayanwale et al. (26). The scale comprises five items: (1) “I lack the necessary computer skills to use AI technology effectively,” (2) “My knowledge and awareness of AI technology are limited,” (3) “I cannot access the Internet, which hinders my ability to explore AI technology,” (4) “I believe that AI may not be beneficial,” and (5) “I struggle to find the time to study and understand AI technology.” This scale has demonstrated satisfactory reliability and validity in prior research, reporting a Cronbach’s *α* of 0.88 (1). Each item was assessed on a 5-point Likert scale (1 = strongly disagree and 5 = strongly agree), with higher total scores indicating a more severe perception of barriers to AI access. The same translation and back-translation procedures were applied to ensure cultural appropriateness (28). Its reliability was further verified in the pilot survey, with a Cronbach’s *α* of 0.748.

### 2.4 Data collection

Data were collected using Wenjuanxing, an online survey platform. Approval was obtained from the relevant hospital departments and nursing administrators prior to data collection. The research team distributed the questionnaire to eligible nurses during their non-working hours via QQ or WeChat. Informed consent was required to participate in the survey. This step was implemented via an online platform that required participants to review and acknowledge the study’s purpose, data confidentiality protocols, and right to withdraw before they could access the survey. Only after they selected “I Agree” could they begin the actual survey. The platform was set to allow only one response per unique IP address and device ID to prevent duplicate submissions.

### 2.5 Data analysis

Statistical analyses were conducted using SPSS 27.0 (IBM Corp.). Descriptive statistics were used to describe the participants’ demographic and professional characteristics, with continuous data presented as means ± standard deviations. Readiness scores were compared across participant subgroups to identify the demographic and professional factors associated with newly qualified nurses’ readiness to adopt AI. The Shapiro–Wilk test was used to assess the normality of continuous variables. Parametric tests were applied for normally distributed data, while non-parametric tests were used when the normality assumption was violated. For multi-category and dichotomous variables, Kruskal–Wallis H and Mann–Whitney U-tests were applied, respectively. Relationships between variables were examined using Spearman’s correlation, and significant factors influencing AI adoption readiness were identified through multiple linear regression. Descriptive statistics were used to analyze the perceived barriers to AI adoption among newly qualified nurses, with statistical significance set at a *p* < 0.05 (two-tailed).

## 3 Results

Table 1 summarizes the participants’ demographic characteristics. The average age of the participants was 24.11 years (SD = 1.482), and the average years of work experience were 1.7 (SD = 0.794). Women comprised 94.2% (*n =* 310) of the sample, and men accounted for only 5.8% (*n =* 19). The majority of participants held a bachelor’s degree (96.4%, *n =* 317), followed by a junior college degree (2.7%, *n =* 9). Regarding the clinical department distribution, the largest proportion of participants worked in internal medicine (44.4%, *n =* 146).

**Table 1:** Demographic characteristics of all participants (*n =* 329).

Variable	Mean (SD)	*n*(%)
Age (years)	24.11(1.482)	
Gender		
Men		19(5.8)
Women		310(94.2)
Education		
Junior college degree		9(2.7)
Bachelor’s degree		317(96.4)
Master’s degree or above		3(0.9)
Years of working		
1		167(50.8)
2		93(28.2)
3		69(21.0)
Department		
Internal medicine		146(44.4)
Surgery		33(10.0)
Gynecology		6(1.8)
Pediatrics		8(2.4)
Emergency		19(5.8)
ICU		14(4.3)
Operating room		24(7.3)
Outpatient clinic		28(8.5)
Other		51(15.5)
AI technical training		
Yes		152(46.2)
No		177(53.8)
Duration of Internet usage		
<1 h		46(14.0)
1–3 h		131(39.8)
3–6 h		86(26.1)
>6 h		66(20.1)
Understanding of AI technology		
Very poor		22(6.7)
Poor		75(22.8)
Fair		191(58.)
Good		22(6.7)
Very good		19(5.8)
Familiarity with AI Technology		
Very poor		26(7.9)
Poor		87(26.4)
Fair		184(55.9)
Good		15(4.6)
Very good		17(5.2)
Awareness of AI in Nursing		
Very poor		31(9.4)
Poor		85(25.8)
Fair		176(53.5)
Good		17(5.2)
Very good		20(6.1)

Table 2 presents the results of the univariate analysis examining the relationship between participant characteristics and readiness to adopt AI among newly qualified nurses. Readiness to adopt AI differed significantly among newly qualified nurses based on whether they had received AI technical training (*z* = −6.997, *p* < 0.001), duration of Internet usage (*H* = 8.352, *p* = 0.039), level of understanding of AI (*H* = 56.138, *p* < 0.001), AI familiarity level (*H* = 61.112, *p* < 0.001), and awareness of AI in nursing (*H* = 64.203, *p* < 0.001).

**Table 2:** Univariate analysis of participant characteristics associated with readiness to adopt AI (*n =* 329).

Variable	Total score of readiness to adopt artificial intelligence, Mean (SD)	*z/H*	*p*
Gender^a^		−0.475	0.635
Men	9.84(1.98)		
Women	9.85(1.97)		
Education^b^		0.425	0.808
Junior college degree	10.00(1.94)		
Bachelor’s degree	9.85(1.97)		
Master’s degree or above	9.33(1.53)		
Years of working^b^		0.797	0.850
1	9.81(1.97)		
2	9.75(1.82)		
3	9.93(1.91)		
Department^b^		5.530	0.700
Internal medicine	9.99(2.05)		
Surgery	9.97(2.05)		
Gynecology	10.50(1.79)		
Pediatrics	9.13(1.46)		
Emergency	9.37(1.95)		
ICU	9.71(2.02)		
Operating room	9.63(1.84)		
Outpatient clinic	10.07(2.36)		
Other	9.63(1.56)		
AI technical training^a^		−6.997	<0.001
Yes	10.63(1.99)		
No	9.18(1.68)		
Duration of Internet usage^b^		8.352	0.039
<1 h	9.50(1.82)		
1–3 h	9.91(1.89)		
3–6 h	9.55(1.67)		
<6 h	10.37(2.44)		
Understanding of AI technology^b^		56.138	<0.001
Very poor	8.77(1.77)		
Poor	9.05(1.86)		
Fair	9.9(1.70)		
Good	11(2.20)		
Very good	12.47(1.90)		
Familiarity with AI Technology^b^		61.112	<0.001
Very poor	8.58(1.70)		
Poor	9.14(1.85)		
Fair	9.99(1.72)		
Good	11.47(1.77)		
Very good	12.53(2.00)		
Awareness of AI in Nursing^b^		64.203	<0.001
Very poor	8.39(1.73)		
Poor	9.24(1.74)		
Fair	9.99(1.74)		
Good	11(1.80)		
Very good	12.5(2.04)		

^a^Mann–Whitney U-test. ^b^Kruskal–Wallis H test.

Table 3 presents descriptive statistics for the key variables. The mean score for readiness to adopt AI was 9.85 (SD = 1.97), which is slightly above the theoretical midpoint of 9. The mean score for perceived barriers to accessing AI was 14.88 (SD = 3.28). The mean score for perceived usefulness in nursing practice was 28.13 (SD = 5.72). The mean perceived ease of use score was 26.55 (SD = 5.49).

**Table 3:** Descriptive summary of the key study variables.

Variable	*α*	Mean	SD
Readiness to adopt AI technology	0.735	9.85	1.97
Perceived barriers to accessing AI technology	0.748	14.88	3.28
Perceived AI usefulness in nursing practice	0.966	28.13	5.72
Perceived ease of use of AI in nursing practice	0.95	26.55	5.49

Table 4 presents the results of Spearman’s correlation analysis of the key variables. Readiness to adopt AI among newly qualified nurses showed a significant positive correlation with perceived ease of use (*r* = 0.291, *p* < 0.01) and perceived usefulness (*r* = 0.206, *p* < 0.01), while demonstrating a significant negative correlation with perceived barriers to accessing AI technology (*r* = −0.19, *p* < 0.01). A strong positive correlation was observed between perceived ease of use and perceived usefulness of AI in nursing practice (*r* = 0.715, *p* < 0.01).

**Table 4:** Spearman’s correlation analysis.

Variable	1	2	3	4
1. Readiness for AI technology	1			
2. Perceived ease of use of AI in nursing practice	0.291**	1		
3. Perceived AI usefulness in nursing practice	0.206**	0.715**	1	
4. Perceived barriers to accessing AI technology	−0.19**	−0.102	−0.028	1

^**^ *p* < 0.01.

Multicollinearity was assessed before conducting regression analysis. The tolerances of all independent variables ranged from 0.193 to 0.964, and the VIF values ranged from 1.037 to 5.189, all of which were far below the critical values, indicating that the model did not have a serious problem with multicollinearity. The residuals of the dependent variable satisfied the normal distribution, independence, conditions of linearity, and homoscedasticity.

Multiple linear stepwise regression analysis revealed that perceived ease of use of AI in nursing practice (*β* = 0.211, SE = 0.027, *p* = 0.006), prior to receiving training (*β* = 0.23, SE = 0.196, *p* < 0.001), and awareness level of AI in nursing (*β* = 0.201, SE = 0.179, *p* = 0.018) emerged as significant predictors of newly qualified nurses’ readiness to adopt AI (Table 5).

**Table 5:** Multiple linear stepwise regression analysis of factors influencing nurses’ readiness to adopt artificial intelligence (*n =* 329).

Variable	B(95%CI)	SE	*β*	*t*	*p*
Perceived barriers to accessing AI technology	−0.003(−0.058,0.053)	0.028	−0.005	−0.096	0.923
Perceived AI usefulness in nursing practice	−0.003(−0.053,0.047)	0.025	−0.008	−0.111	0.912
Perceived ease of use of AI in nursing practice	0.075(0.022,0.129)	0.027	0.211	2.762	0.006
AI technical training	0.905(0.520,1.290)	0.196	0.230	4.627	<0.001
Duration of Internet usage	−0.08(−0.272,0.112)	0.098	−0.039	−0.82	0.413
Understanding of AI technology	0.072(−0.356,0.500)	0.217	0.032	0.331	0.741
Familiarity with AI technology	0.312(−0.156,0.780)	0.238	0.138	1.312	0.191
Awareness of AI in nursing	0.426(0.073,0.779)	0.179	0.201	2.374	0.018

The descriptive analysis identified the main perceived barriers to AI adoption among newly qualified nurses (Table 6). More than 50% of participants (51.4%) reported a lack of necessary knowledge and awareness of AI technology, with 43.2% agreeing and 8.2% strongly agreeing. Similarly, over 45% of participants indicated insufficient computer skills to effectively use AI technology, with 38.1% agreeing and 7.9% strongly agreeing. Time constraints presented another significant barrier, with 34.6% reporting insufficient time to use AI technologies (28.8% agreeing, 5.8% strongly agreeing). Regarding infrastructure access, 42.3% of participants reported being able to access the Internet, while 24.6% lacked Internet access. Notably, a substantial majority (70.8%) disagreed that AI might not be beneficial, reflecting generally positive attitudes toward AI. This study revealed that the most prominent barriers to AI adoption include limited AI knowledge and awareness, inadequate computer skills, and time constraints for learning.

**Table 6:** Descriptive analysis of perceived barriers to accessing AI technology (frequency, %).

Items	Strongly disagree	Disagree	*N*eutral	Agree	Strongly agree	Mean	Rank
1. I lack the necessary computer skills to effectively utilize AI technology.	10(3)	34(10.3)	134(40.7)	125(38.1)	26(7.9)	3.37	2
2. My knowledge and awareness of AI technology are limited.	8(2.4)	24(7.3)	128(38.9)	142(43.2)	27(8.2)	3.47	1
3. I cannot access the internet, which hinders my ability to explore AI technology.	39(11.9)	100(30.4)	109(33.1)	68(20.6)	13(4.0)	2.74	4
4. I believe that AI may not be beneficial.	70(21.3)	163(49.5)	72(21.9)	16(4.9)	8(2.4)	2.18	5
5. I struggle to find the time to study and understand AI technology.	22(6.7)	53(16.1)	140(42.6)	95(28.8)	19(5.8)	3.11	3

## 4 Discussion

This study assessed AI readiness among newly qualified nurses and explored associated factors. The mean readiness score of 9.85 (SD = 1.97) was slightly above the theoretical midpoint of 9, indicating a moderate level of readiness for AI adoption. This suggests that newly qualified nurses demonstrate a willingness to integrate AI technologies into their clinical practice and professional development, with the potential for further enhancement through appropriate guidance and support. These findings align with previous research, indicating that newly qualified nurses can recognize the potential advantages and value of AI technology in the nursing field and are willing to explore the application of AI in their clinical practice and career development (13, 32, 33).

Multiple linear regression analysis identified key factors influencing newly qualified nurses’ readiness to adopt AI technology. These findings strongly support the applicability of the TAM in this specific population. Perceived ease of use emerged as a significant positive predictor of AI readiness, indicating that the simplicity and usability of AI systems are critical factors for newly qualified nurses who must simultaneously adapt to clinical responsibilities and new technologies (34). This suggests that even potentially beneficial AI tools may face rejection if they are perceived as complex or difficult to master (35).

This study identified prior AI training as a significant positive predictor of readiness among newly qualified nurses, with those who had received training demonstrating significantly higher readiness scores than their untrained counterparts. This may be attributed to structured and systematic training that effectively addresses knowledge and skill gaps in AI, thereby enhancing nurses’ confidence and willingness to use these technologies (36).

The study further revealed that understanding of AI technology was significantly associated with AI readiness, suggesting that clearer comprehension of AI’s capabilities enhances newly qualified nurses’ preparedness to use it in clinical settings. Similarly, familiarity with AI technologies demonstrated a significant relationship with readiness levels. Nurses with greater familiarity are likely to experience reduced uncertainty and lower anxiety regarding AI adoption, thereby developing higher readiness (37). Furthermore, awareness of AI’s role in nursing emerged as a significant factor influencing readiness, which is consistent with previous research (20). This indicates that successful AI integration may depend more on cultivating positive perceptions of and trust in the value of AI than on technical understanding alone (38). When newly qualified nurses perceive AI as a collaborative partner in nursing advancement rather than a threat, they demonstrate greater willingness to overcome challenges and engage with these technologies.

This study found that perceived usefulness was not a significant predictor of newly qualified nurses’ readiness to adopt AI, contrary to predictions by the TAM. This is consistent with Rony (39), who suggested that individuals with limited experience may lack sufficient opportunities to use and integrate AI tools into their work, making it difficult to form stable beliefs about their usefulness. For newly qualified nurses, this may be attributed to their status as novices in clinical practice, who are primarily engaged in skill acquisition and role adaptation. Their main focus is on mastering basic clinical tasks and adapting to a fast-paced working environment (35), thereby limiting their opportunities to deeply integrate AI tools. Thus, they cannot establish stable perceptions of their usefulness. Therefore, providing newly qualified nurses with opportunities to engage with AI tools in real clinical scenarios during their clinical adaptation period may help them develop a clearer and more stable understanding of AI’s practical value.

The study found that perceived barriers did not significantly affect AI readiness, which is consistent with previous studies (1). This may be because newly qualified nurses’ readiness to use AI is more strongly influenced by positive factors such as perceived ease of use, prior AI training, and awareness of AI. However, the lack of statistical significance does not diminish the actual importance of these reported obstacles. They remain prevalent and cannot be overlooked. “Limited AI knowledge and awareness” and “lack of necessary computer skills” were identified as the two most prominent obstacles, ranking first and second, respectively, in frequency (1). Notably, “lack of time” emerged as the third most reported barrier in this study, which differs from earlier findings. This discrepancy may be attributed to the demanding clinical responsibilities and fast-paced environments that newly qualified nurses encounter upon entering practice, leaving limited capacity for learning new technologies (40). These findings highlight the importance of integrating AI training into onboarding programs and ensuring sufficient time and resources to support new nurses in adopting AI effectively.

This study extended the TAM by incorporating the variable of perceived barriers and applied this framework to investigate newly qualified nurses’ readiness to adopt AI. The findings not only reaffirm the crucial role of perceived ease of use but also demonstrate that positive awareness of AI and effective training collectively influence AI readiness in this population. Although perceived barriers to accessing AI technology did not emerge as statistically significant predictors in the regression model, their widespread prevalence indicates that they constitute a crucial dimension that must be considered. These barriers represent specific challenges that must be systematically addressed in future AI training programs and implementation strategies.

### 4.1 Implications

The findings of this study provide profound insights into promoting the acceptance and integration of AI technologies by newly qualified nurses. First, nursing managers should consider integrating AI technology training into newly qualified nurses’ onboard education and designing systematic AI-related special courses (41). Training programs should prioritize the development of technical skills and capabilities among newly qualified nurses, emphasizing their technical literacy and operational proficiency (42). Furthermore, incorporating real-world cases in which AI supports clinical decision-making and nursing tasks during training may deepen nurses’ understanding of the value of AI technology and thereby enhance their readiness to adopt it (1, 43).

### 4.2 Limitations

This study had several limitations. First, the cross-sectional design limits causal inferences between the examined variables and nurses’ AI readiness. Future research should include longitudinal or intervention studies to clarify these dynamics. Second, self-reported measures may introduce certain biases, and introducing qualitative approaches in future investigations could provide a deeper understanding of the complex perceptions of newly qualified nurses regarding AI. Finally, the use of convenience sampling from four tertiary Grade-A hospitals in Shandong Province may limit the generalizability of the findings to other nursing populations and healthcare settings.

## 5 Conclusion

This study revealed a moderate level of readiness for AI adoption among newly qualified nurses. Their readiness was significantly influenced by perceived ease of use, prior AI training, and AI awareness. Although perceived barriers did not emerge as a significant predictor in the regression model, they remain a noteworthy factor, with moderate obstacles identified in this population. By incorporating the variable of perceived barriers, this study expands the classic TAM, confirming its applicability while simultaneously highlighting the need for contextual adaptation when applying theoretical frameworks to complex healthcare environments. These findings provide a foundation for developing targeted strategies to enhance AI integration in nursing practice.

## Data Availability

The original contributions presented in the study are included in the article/supplementary material, further inquiries can be directed to the corresponding author.
