# Correction: Artificial intelligence readiness and its influencing factors among newly qualified nurses: a cross-sectional study

**DOI:** 10.3389/fmed.2026.1798340

**Published:** 2026-02-11

**Authors:** Qianqian Yang, Min Zhao, Linlin Yang, Xiaobing Wang, Chunling Yang

**Affiliations:** 1 Department of Nursing, Liaocheng People's Hospital, Medical School of Liaocheng University, Liaocheng, Shandong, China; 2 School of Nursing and Rehabilitation, Shandong University, Jinan, Shandong, China; 3 School of Nursing, Shandong First Medical University, Shandong Academy of Medical Sciences, Tai'an, Shandong, China; 4 Department of Nursing, Liaocheng People's Hospital, Liaocheng, Shandong, China; 5 Obstetrics Department, Liaocheng People's Hospital, Liaocheng, Shandong, China

**Keywords:** artificial intelligence readiness, newly qualified nurses, perceived barriers, perceived ease of use, technology acceptance model

Author Chunling Yang was erroneously assigned to affiliation School of Nursing and Rehabilitation, Shandong University, Jinan, Shandong, China. This affiliation has now been removed for author Chunling Yang.

Affiliation Department of Nursing, Liaocheng People's Hospital, Liaocheng, Shandong, China was erroneously given as Nursing Department of Lioocheng People's Hospital, Liaocheng, Shandong, China.

Affiliation Department of Nursing, Liaocheng People's Hospital, Medical School of Liaocheng University, Liaocheng, Shandong, China was erroneously given as Liaocheng People's Hospital, Medical School, Liaocheng University, Liaocheng, Shandong, China.

The original version of this article has been updated.

